# Effects of Excision of the Syphilitic Chancre

**Published:** 1881-10

**Authors:** 


### Effects of Excision of the Syphilitic Chancre.

M. Mauriac reports (*Gazette des Hopitaux*, 1881, No. 7, 10, 14) seven carefully recorded cases in which he excised the initial lesion of syphilis. In six, excision was performed at periods varying from four to sixteen or eighteen days after the appearance of the sore. In the seventh case, the initial lesion was excised about fifty hours after it had been first noticed, and before there was the least trace of glandular enlargement; but in this, as well as in all the others, the operation was unsuccessful in preventing further development of the disease.—*London Med. Record, June 15, 1881.*

